# Eucalyptus essential oil exerted a sedative-hypnotic effect by influencing brain neurotransmitters and gut microbes via the gut microbiota-brain axis

**DOI:** 10.3389/fphar.2024.1464654

**Published:** 2024-09-25

**Authors:** Xuejiao Li, Yuanyi Zhang, Qian Zhang, Aizhi Cao, Jie Feng

**Affiliations:** ^1^ Key Laboratory of Animal Feed and Nutrition of Zhejiang Province, College of Animal Sciences, Zhejiang University, Hangzhou, China; ^2^ Biotechnology R&D Center of Shandong Longchang Animal Health Products Co., Ltd., Jinan, China

**Keywords:** eucalyptus essential oil, sedative-hypnotic, neurotransmitter, gut microbes, gut microbiota-brain axis

## Abstract

Sleep disorders are becoming more and more common, leading to many health problems. However, most of current available medications to treat sleep disorders are addictive and even impair cognitive abilities. Therefore, it is important to find a natural and safe alternative to treat sleep disorders. In this study, twenty-four 8-week-old male ICR mice (25 ± 2 g) were equally divided into three groups: the control group (gavage of 0.9% saline), the eucalyptus essential oil (EEO) group (10 mg/kg B.W.), and the diazepam group (1 mg/kg B.W.). Firstly, open field test and sleep induction test were used to determine the sedative-hypnotic effect of EEO. Secondly, the effect of EEO on neurotransmitters in the mice brain was determined. Finally, based on the gut microbiota-brain axis (GMBA), the effect of EEO on the intestinal flora of mice was explored. It was found that EEO significantly reduce the activity and prolong the sleep duration of mice, exhibiting a good sedative-hypnotic effect. In the brain, EEO could increase the levels of sleep-promoting neurotransmitters, such as glutamine, Gamma-aminobutyric acid (GABA), glycine, tryptophan, N-acetylserotonin, and 5-hydroxyindoleacetic acid (5-HIAA). In the intestine, EEO was found to increase the diversity of gut microbes, the abundance of short chain fatty acid (SCFA) producing flora, and the abundance of functional flora synthesizing GABA and glycine neurotransmitters. These studies suggested that EEO exerted a sedative-hypnotic effect by acting on gut microbes and neurotransmitters in the brain. EEO has the potential to become a natural and safe alternative to traditional hypnotic sedative drugs.

## Introduction

Insomnia can seriously influence people quality of life, and recent studies have revealed its association with obesity, diabetes, heart disease, coronary artery disease, depression and other serious problems (Walia and Mehra, 2016; Broussard et al., 2016). It is estimated that 50–70 million American adults suffer from one or more sleep disorders (Harding and Feldman, 2008), so does 31% of Western Europeans and 23% of Japanese (Dasdelen et al., 2022). In recent decades, a large number of medications have appeared on the market for the treatment of various types of sleep disorders such as chloral hydrate, barbiturates, benzodiazepine, benzodiazepine agonists, modafinil, and antidepressants. However, these medications can cause dependence even addiction, and some may even gradually impair cognitive abilities (Matheson and Hainer, 2017). Therefore, it is important to find safe and effective substances to replace existing commercially available medications to treat sleep disorders.

Eucalyptus essential oil (EEO) is extracted from eucalyptus branches and leaves by steam distillation and has a wide range of applications. The main components of EEO are 1,8-eudesmusin and terpineol (Ieri et al., 2019). Many of the components of EEO (Bampidis et al., 2023) and EEO (Regulation (EC) No 1831/2003) are authorized as a food additive and animal feed additive. EEO has been reported to have antibacterial, anti-inflammatory, insecticidal, antioxidant, analgesic, and other excellent biological activities (Caceres et al., 2017; Aziz et al., 2019; Barbosa et al., 2021; Sathantriphop et al., 2014). It has been found that 1,8-eudesmusin can act on the nervous system, affecting the synthesis of neurotransmitters and then exerting analgesic effects (Takaishi et al., 2012). A study has shown that 1,8-eudesmusin can also inhibit locomotion in mice, showing a good sedative effect (Santos and Rao, 2000). However, the sedative-hypnotic effect of EEO has not been studied thoroughly and systematically.

Neurotransmitters in the central nervous system play an important role in the regulation of the sleep-wake cycle as key mediators of neuronal signal (Ngo and Vo, 2019). In recent years, the gut microbiota-brain axis (GMBA) has been a hot topic in biomedical researches and suggested as a potential therapeutic target for central nervous system disorders (Pesarico et al., 2023). Gut microbes not only affect the digestive, metabolic, and immune functions of the host, but also regulate the sleep state of the host through the GMBA (Poroyko et al., 2016; Golubeva et al., 2017; Niu et al., 2022). High-quality sleep has been found to be closely related to the composition of gut microbes, and in addition, gut microbes dysbiosis can exacerbate the cognitive dysfunction in sleep-deprived mice (Torres-Fuentes et al., 2017; Li N. et al., 2023). Gut microbes have been shown to be involved in the production of a variety of neurotransmitters and cytokines (Galland, 2014). Besides, gut microbes and their metabolites may also influence the expression of genes associated with the biological clock in the central nervous system and liver, then affecting sleep quality (Leone et al., 2015). It is necessary to study how EEO works on neurotransmitters and gut microbes for deep understanding the observed improvement in sleep quality. Therefore, the study was conducted to investigate the effects of EEO on brain neurotransmitters and gut microbes in mice through GMBA to find the possible targets of EEO for the treatment of sleep disorders.

## Methods

### Animals and drugs

All animal testing procedures were conducted in accordance with the guidelines of the Animal Ethics Committee of Zhejiang University (ethical programme code ZJU 20160403). A total of twenty-four 8-week-old male ICR mice (25 ± 2 g) were purchased from Shanghai Lingchang for this study. The 24 mice were equally divided into three groups: the control group (gavage of 0.9% saline), the EEO group (10 mg/kg B.W.), and the diazepam group (1 mg/kg B.W.). Daily gavage was administered at 9:00 a.m. for 14 days. The test environment was controlled at 20°C–25°C and 40%–70% relative humidity. Acclimatisation in the animal house had been carried out for 1 week till the test. EEO was provided by Shandong Longchang Animal Health Products Co. Diazepam was purchased from Shanghai Xudong Haipu Pharmaceutical Co., Ltd., Batch No. H13021864. Pentobarbital sodium salt (CAS no: 57-33-0) was purchased from Sigma-Aldrich, United States.

### Preparation of EEO

The method of preparing EEO refers to CN 110558324B. Australian eucalyptus leaves were cleaned, dried at 180°C and then crushed, and the eucalyptus oil was obtained by using water vapour distillation, and finally the eucalyptus oil was added to a vacuum distillation column for distillation to remove impurities, resulting in EEO. Measure out 100 mg of EEO and mix it with 1 mL of Tween 80, then dissolve the mixture in 49 mL of 0.9% saline solution. Shake the mixture thoroughly using a shaker, and store the EEO in a sealed, light-proof container in a refrigerator at 4°C for future experimental use. At this point, the concentration of EEO was 2 mg/mL. The EEO of 2 mg/mL is used for subsequent mice gavage experiment.

### Open field test

Thirty min following the final drug administration to the mice in each group, the mice were placed into the open field with a space of 50 cm^3^ × 50 cm^3^ × 40 cm^3^, made of acrylic sheet and aluminium alloy frame, where the photographic equipment (model 550D, Canon digital camera) was suspended at 2 m directly above. After 5 min of acclimatisation, the activities of the mice during the 5 min were recorded. Trajectory analysis was performed by the video analysis system (SANS, SA215).

### Sleep induction test

The sleep induction test was referred to the Medeiros study (Medeiros et al., 2018). After the last treatment, all mice were injected intraperitoneally with sodium pentobarbital solution salt according to 50 mg/kg B.W. standard. Sleep onset was determined by the disappearance of reflex for 1 min after administration of pentobarbital sodium salt solution. Sleep latency was the time from the injection of the drug to the disappearance of the reflex, and sleep duration was the time from the disappearance of the reflex to its appearance. The sleep latency and sleep duration were recorded for each group.

### Measurement of neurotransmitter levels

Liquid chromatography tandem mass spectrometry (LC-MS/MS) was used to determine the content of neurotransmitters in mouse brain (Horvath et al., 2022). The mouse brain was homogenised and extracted with 20% acetonitrile in methanol for 3 min, then centrifuged (12,000 × g, 4°C, 10 min), and the supernatant was refrigerated at −20°C for 30 min, then centrifuged again, the supernatant was taken as the test solution. The chromatographic column used was a Waters ACQUITY UPLCC HSS T3 C18 column (1.8 μm, 100 mm × 2.1 mm i.d.), and the column temperature was controlled at 40°C. Mobile phases A and B were ultrapure water (containing 0.1% formic acid) and acetonitrile (containing 0.1% formic acid), respectively, and the control flow rate was 0.35 mL/min. The mass spectrometry conditions were based on the use of an electrospray ionization source, with the mass spectral voltage (MSV) set to 5,500 V in the positive ion mode and to −4,500 V in the negative ion mode.

### Determination of gut microbes

The mice were dissected at the end of the test and the contents of their cecum were collected in sterile conical tubes. DNA was extracted from the cecal content using the CTAB method according to manufacturer’s instructions. The V3 to V4 regions of the microbiota 16S rDNA gene were amplified using 338F/806R primers, then the amplicon was purified using the Qiagen gel extraction kit (Qiagen, Hilden, Germany) and sequenced by the Illumina HiSeq 2500 PE250 platform (Novogene Bioinformatics Technology Co., Ltd., Tianjin, China). Analysis and visualization of high-throughput sequencing data were collected via the online Novomagic Cloud Platform.

### Statistical analysis

Data were tested for significance using one-way ANOVA in SPSS version 22.0 software (IBM, United States), and data were expressed as mean ± standard deviation (M ± SD), with *P < 0.05 being significant and **P < 0.01 being highly significant. Other statistical methods were detailed in the relevant figures.

## Results

### Effects of EEO on locomotion in mice

**FIGURE 1:**
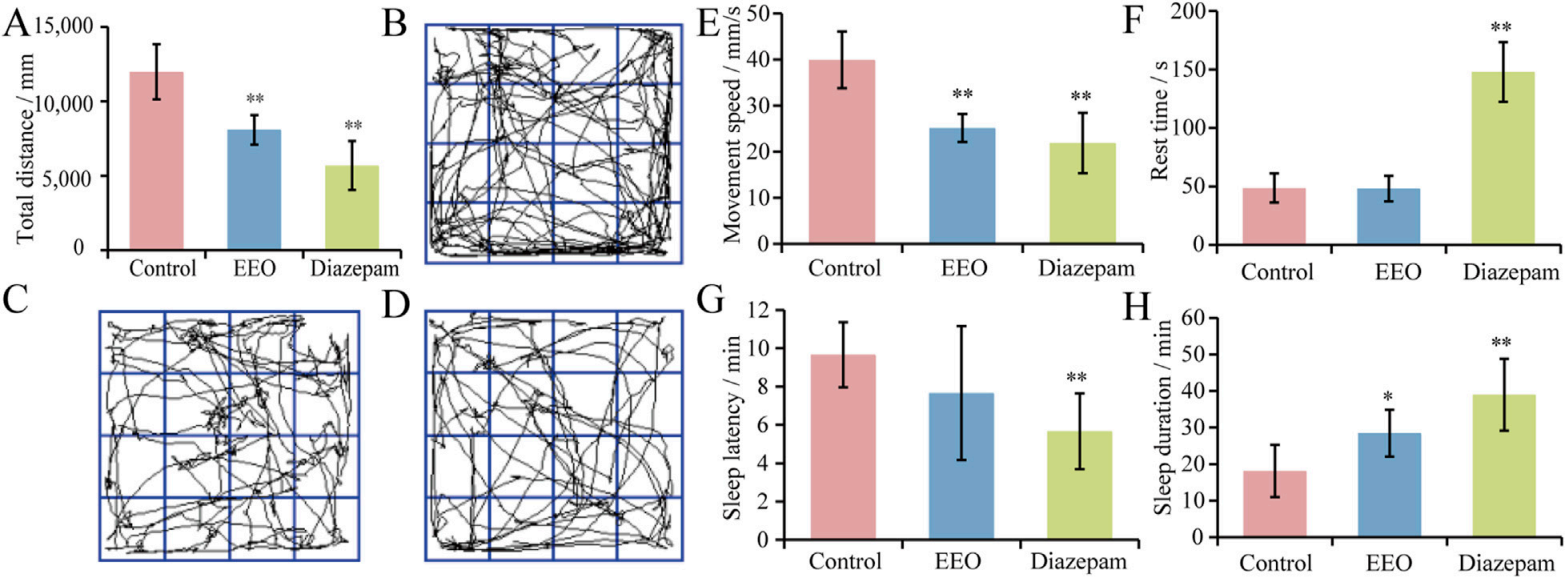
Effects of EEO on the behaviors of mice. **(A)** Total distance. **(B–D)** The behavioural trajectories of mice in the control group, EEO group, and diazepam group, respectively. **(E)** Movement speed. **(F)** Rest time. **(G)** Sleep latency. **(H)** Sleep duration. *p < 0.05, **p < 0.01; n = 7–8.

The effects of EEO on the total distance, movement speed and rest time of mice were shown in Figures 1A, E, F. Compared with the control group, the total distance travelled and the speed of movement of mice in the EEO group and the diazepam group were highly significantly reduced (*P* < 0.01), with a reduction in the total distance travelled of 32.5% and 52.5%, and the speed of movement was reduced to 25.13 ± 3.01 and 21.88 ± 6.55 mm/s. Figures 1B–D showed the activity trajectories of mice in the control group, EEO group, and diazepam group, respectively, from which it can also be observed that both EEO and diazepam reduced the activity of mice. In terms of resting time, EEO had no significant effect on the resting time of mice (*P* > 0.05), however, diazepam significantly increased the resting time of mice (*P* < 0.01).

### Effects of EEO on sleep in mice

The effects of EEO on sleep latency and sleep duration in mice were shown in Figures 1G, H. Gavage of EEO resulted in a shortening of sleep latency time, but there was no significant difference, and diazepam reduced the sleep latency of mice with a highly significantly (*P* < 0.01). The sleep duration of mice in control, EEO and diazepam groups were 18.17 ± 7.15, 28.50 ± 6.37 and 39.00 ± 9.85 min, respectively. Compared with the control group, EEO significantly increased the sleep duration of mice (*P* < 0.05), diazepam highly significantly increased the sleep duration of mice (*P* < 0.01). EEO and diazepam prolonged the sleep duration of mice by 56.9% (*P* < 0.05) and 114.7% (*P* < 0.01), respectively.

### Effects of EEO on neurotransmitters in the mouse brain

**FIGURE 2:**
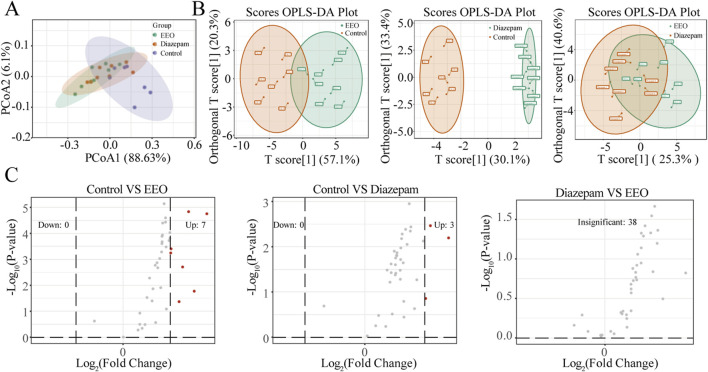
Effects of EEO gavage on neurotransmitters diversities of mice. **(A)** Principal coordinate analysis (PCoA) of weighted UniFrac distance. PCoA was performed by statistics function ggplot2 within R (http://www.r-project.org). **(B)** Orthogonal Partial Least Squares-Discriminant Analysis (OPLS-DA). After pre-processing the original data in OPLS-DA by $X^{*}=X-\bar{X}$ , the OPLSR. Anal function in the MetaboAnalystR package in R software is used for analysis. **(C)** Volcano plot between different groups. Using fold change analysis, if fold change of a neurotransmitter is ≥2 or ≤0.5 between the control group and the experimental group, it is considered significantly different. n = 8.

We analysed the effects of EEO on neurotransmitters in the mouse brain. As shown in the PCoA and OPLS-DA score plots, compared with the control group, EEO or diazepam treatment significantly altered neurotransmitters in the mouse brain. The neurotransmitter profile in the brain of mice in the EEO group was similar to that of the diazepam group, with a greater difference from the control group (Figures 2A, B). In addition, volcano plot revealed neurotransmitters that differed significantly between groups (Figure 2C). EEO and diazepam treatments significantly elevated the levels of 7 and 3 neurotransmitters, respectively, compared to the control group. There was no significant difference in neurotransmitter levels between the EEO and diazepam groups.

**FIGURE 3:**
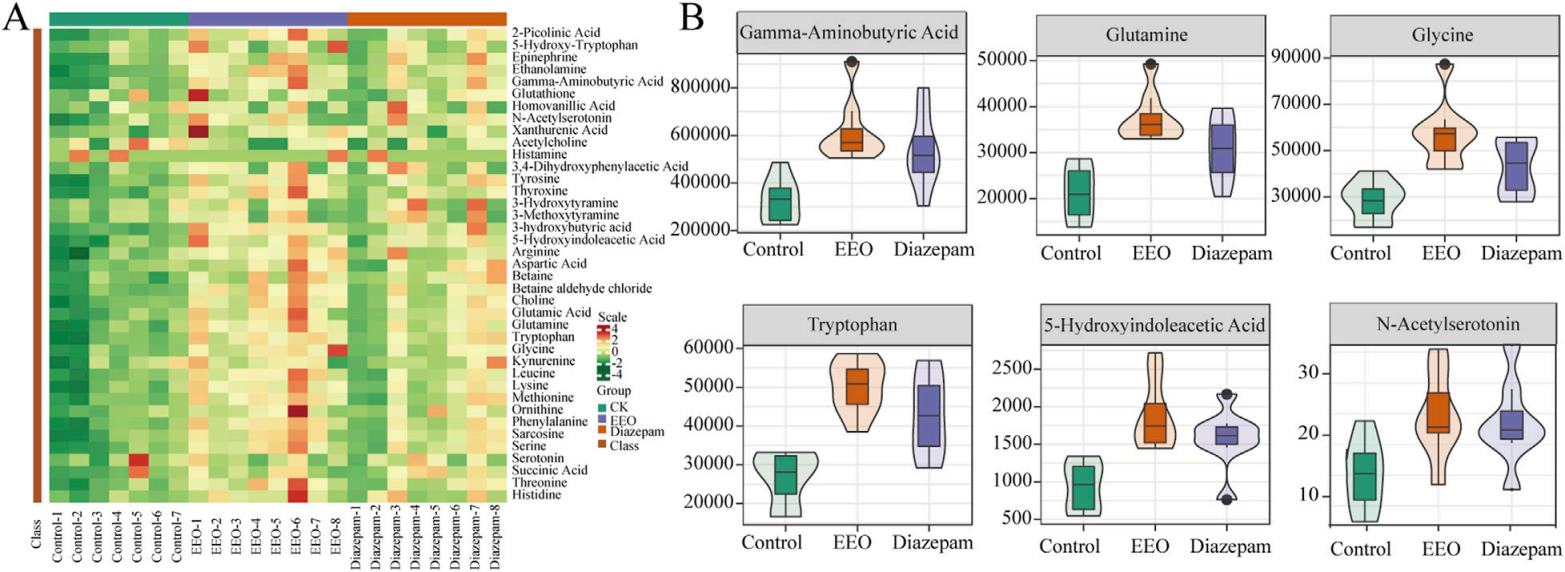
Effects of EEO supplementation on the levels of various neurotransmitters in the mice. **(A)** Heat map of neurotransmitter in different groups. The original levels of differential neurotransmitters are normalized using Unit Variance Scaling and heat maps are generated using R software. **(B)** Neurotransmitters with group differences. Using Variable Importance in Projection (VIP) based on the OPLS-DA model for differential testing, and neurotransmitters considered to be significant if VIP > 0.8. n = 8.

The changes of neurotransmitters were observed after EEO treatment through a clustering heat map (Figure 3A). Brain levels of Gamma-aminobutyric acid (GABA), glutamine, glycine, tryptophan, 5-hydroxyindoleacetic acid (5-HIAA) and N-acetylserotonin were significantly higher in the EEO-treated and diazepam-treated groups of mice than in the control group (Figure 3B).

### Effect of EEO on the diversity of gut microbes

**FIGURE 4:**
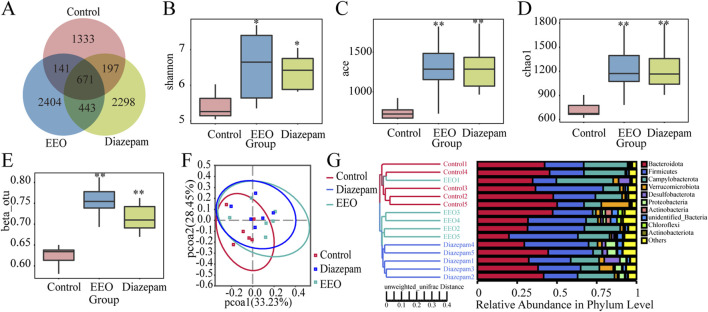
Effects of EEO on the diversity of microbial communities in the cecum of mice. **(A)** Wayne plots at ou level. **(B–D)** Box plots of α-diversity indices: Shannon **(B)**, ACE **(C)** and Chao1 **(D)**. **(E)** β-Diversity indices based on otu level. Species diversity difference is analyzed using Tukey and Kruskal–Wallis rank sum tests. *p < 0.05, **p < 0.01. **(F)** Scatter plots obtained from PCoA based on the weighted UniFrac distance metric. **(G)** UPGMA clustering tree and community structure map based on unweighted UniFrac distance. n = 5–6.

As shown in Figure 4A, the OTUs of mice intestinal flora in the control, EEO and diazepam groups were 2,342, 3,659 and 3,609, EEO and diazepam treatments increased the OTUs of mice intestinal flora, EEO and diazepam groups had more concurrent OTUs (1,114) than the control group duplicated with EEO (812) and diazepam (868) groups, respectively. The α diversity index including shannon, ace and chao1 results were shown in Figures 4B–D. Both EEO and diazepam significantly increased the shannon, ace and chao1 indices of the intestinal microbes in mice compared to the control group (*P* < 0.05). The β diversity index results showed that EEO and diazepam highly significantly increased the intestinal flora of the mouse β diversity (Figure 4E, *P* < 0.01). Based on the weighted UniFrac distance metric, the community composition of each sample was analysed for similarity using principal co-ordinates analysis (PCoA). The microbial communities in the EEO and diazepam groups were altered compared with the control group, and the microbial community species composition was structurally similar in the EEO and diazepam groups (Figure 4F). The similarity between individuals was analysed using the Unweighted UniFrac clustering tree based on the UPGMA method (Figure 4G), and in agreement with the results of the PCoA analysis, the gut microbial community composition in the EEO and diazepam groups was dramatically different compared to the control group.

### Effect of EEO on the relative abundance of gut microbes

**FIGURE 5:**
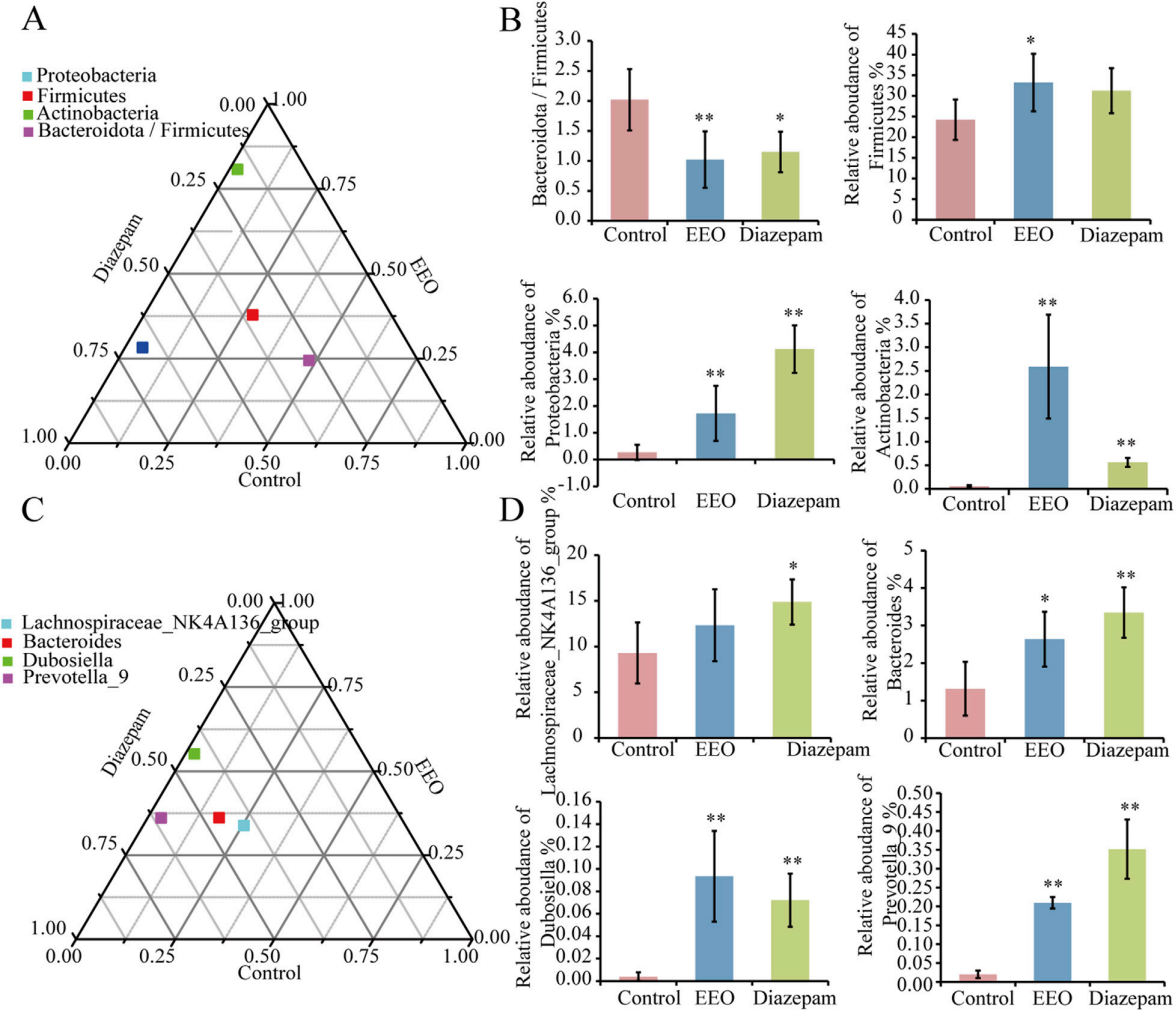
Differences in the abundance of intestinal flora in each group at the phylum and genus levels. Ternary phase diagrams of the differences in intestinal flora at the phylum **(A)** and genus **(C)** levels. **(B)** Histograms of the abundance of intestinal flora at the phylum level in each group after treatment with EEO. **(D)** Histograms of the abundance of intestinal flora at the genus level in each group after eucalyptus essential oils treatment. Find significantly different microbes through t-test analysis. *p < 0.05, **p < 0.01; n = 6.

Differences in dominant species among the three groups of samples at the phylum level are shown in Figure 5A, the study found that the relative abundance of Firmicutes, Proteobacteria, Actinobacteria, and Bacteroidota/Firmicutes differed among the three groups. EEO significantly increased the relative abundance of relative abundance of Firmicutes, Proteobacteria and Actinobacteria (*P* < 0.01) and decreased the abundance ratio of Bacteroidota/Firmicutes (Figure 5B). At the genus level, EEO and diazepam treatment altered the relative abundance of *Bacteroides*, *Dubosiella*, *Prevotella_9*, and *Lachnospiraceae_NK4A136_groups* in the gut microbial community of mice (Figure 5C). EEO and diazepam significantly increased the elevated relative abundance of *Lachnospiraceae_NK4A136_group*, *Bacteroides*, *Dubosiella*, *Prevotella_9* (Figure 5D).

**FIGURE 6:**
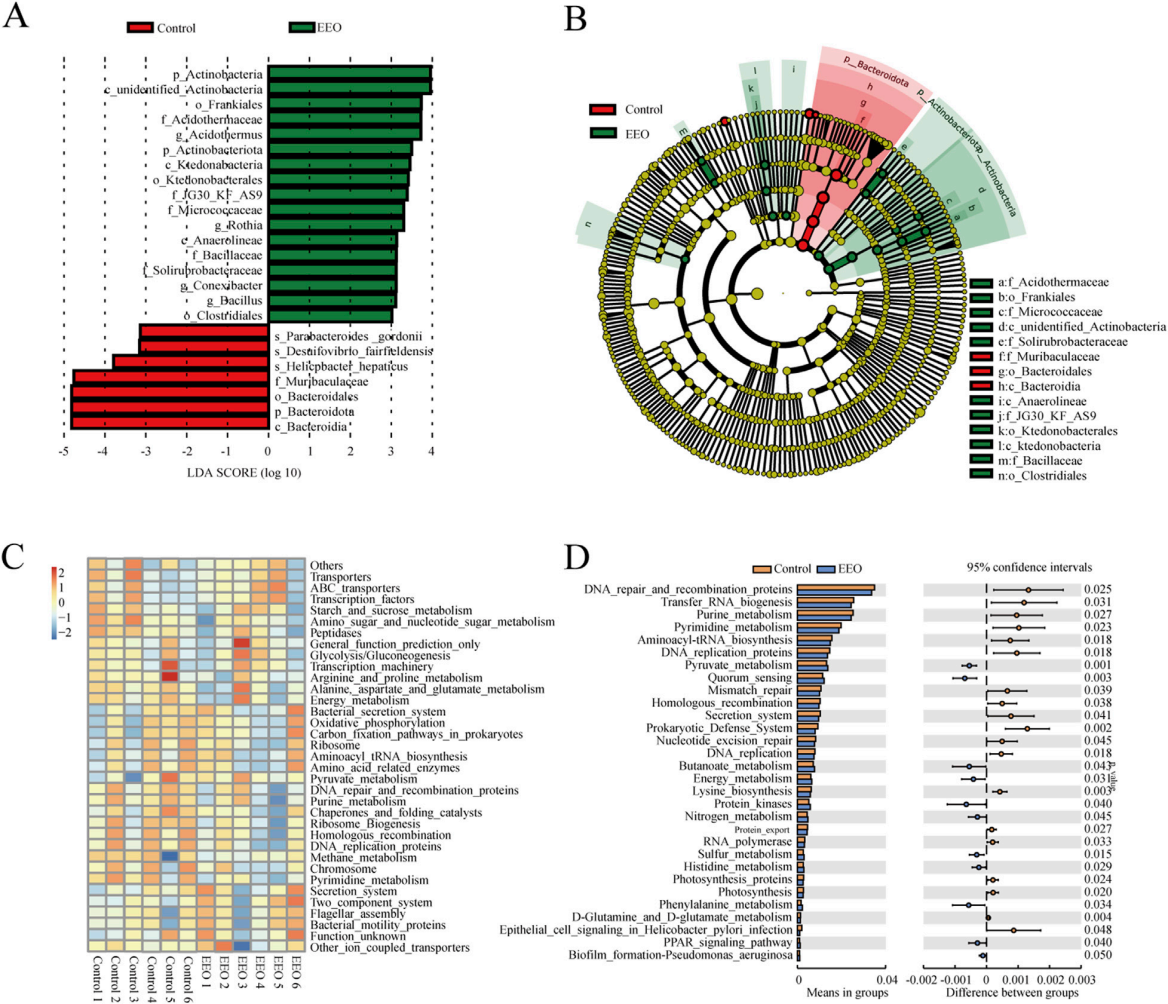
Distinct taxa with greater contributions at different taxonomic levels and the predicted functional changes in the gut microbes. **(A)** Linear discriminant analysis (LDA) effect size (LEfSe) identified microbial biomarkers from phylum to species levels that discriminate the control and EEO groups. **(B)** Cladogram constructed using the LEfSe method to display the phylogenetic distribution of bacteria that were most highly enriched in the control and EEO groups. **(C)** For predicting flora function, heat map of PICRUST analysis identified the KEGG pathway among the control and EEO groups. **(D)** The KEGG analysis showed the potential pathways associated with changes in the intestinal microbes after the EEO treatment. n = 6.

Linear discriminant analysis of LDA effect size (LEfSe) was used to identify taxa most likely to explain differences in microbiota composition between the control and EEO groups. A comparison of the individuals from both groups revealed that Actinobacteria at phylum level, as well as *Acidothermus*, *Rothia*, *Conexibacter* and *Bacillus* at the genus level exhibited significant influences in the EEO group, while in the control, Bacteroidota in the phylum level made contributions (Figure 6A). A taxonomic cladogram derived from the LEfSe analysis (with the LDA score > 3.0) showed the relationships of the differential microbiota with significant roles (Figure 6B). Altogether, these results suggest that the gut microbes exhibit alterations after EEO treatment.

A KEGG functional prediction was subsequently performed by means of the PICRUST approach. The heat map showed that the results of KEGG three-level classification enrichment analysis (Figure 6C). The results showed that these differential microbes might be closely related with purine and pyrimidine metabolism, aminoacyl tRNA biosynthesis, energy metabolism and glutamine and glutamate metabolism (Figure 6D).

### Correlation analysis between gut microbes and sedation behavior

**FIGURE 7:**
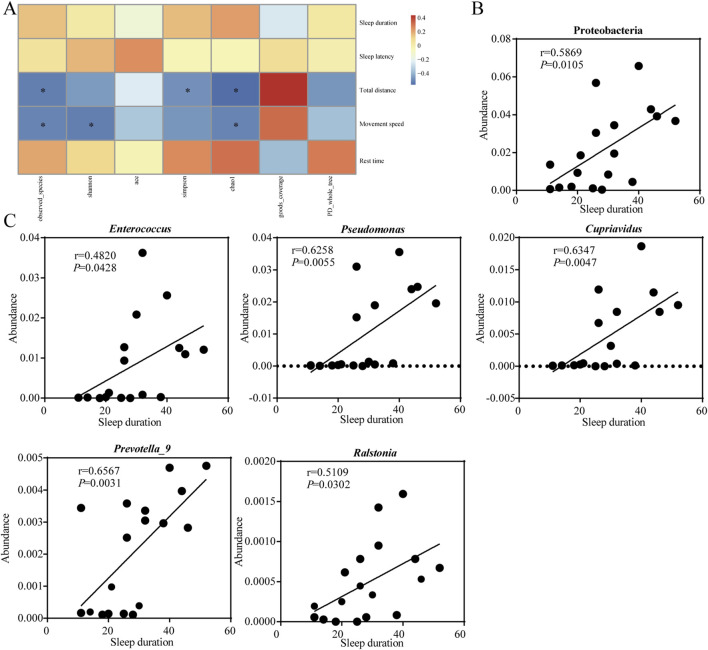
Sedative-hypnotic behaviors are associated with occupancy in the intestinal microbes. **(A)** Correlation analysis between mice behaviors with intestinal microbes α diversity. **(B)** Correlation analysis between sleep duration time and the abundance of gut microbes at the phylum level. **(C)** Correlation analysis between sleep duration time and the abundance of gut microbes at the genus level. Statistical significance is evaluated by Pearson correlation coefficient test. *p < 0.05; n = 18.

The correlation and significance between gut microbes and sedation behaviors in mice was investigated by Pearson’s correlation analysis (Figure 7A). The observed species, chao1 index showed significantly negative correlation (*P* < 0.05) with total distance and movement speed of mice. Shannon, simpson index showed significantly negative correlation (*P* < 0.05) with movement speed and total distance travelled respectively. Sleep duration was positively correlated with the abundance of Proteobacteria at the phylum level (Figure 7B), and positively correlated with *Enterococcus*, *Pseudomonas*, *Cupriavidus*, *Prevotella_9* and *Ralstonia* at the genus level (Figure 7C). It suggested that the length of sleep duration was related to the composition of the gut microbes.

### Effect of EEO on the abundance of neurotransmitter-producing microbes

**FIGURE 8:**
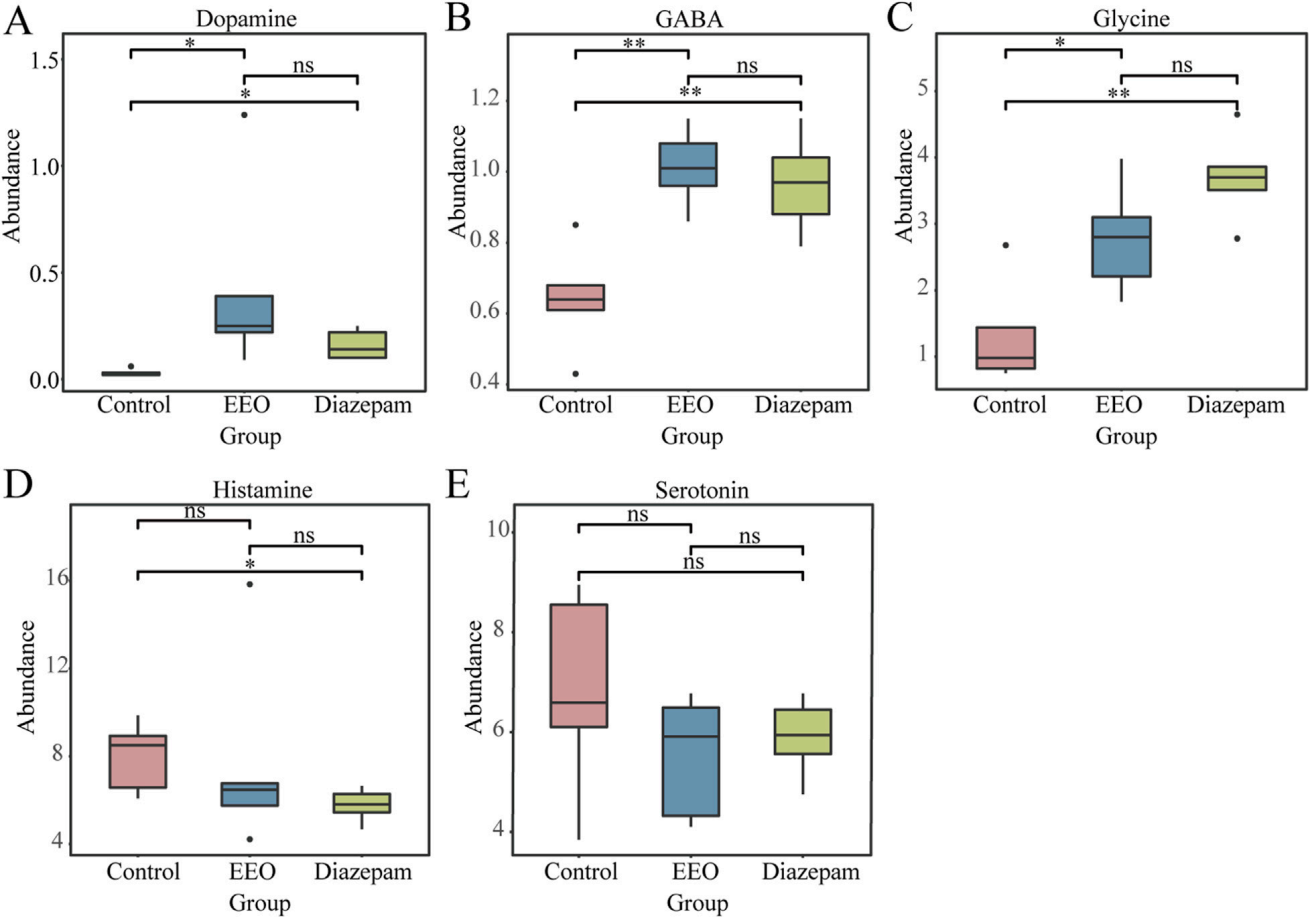
Effects of EEO supplementation on the intestinal neurotransmitter-producing flora of mice. The abundance of functional microbes synthesizing dopamine **(A)**, GABA **(B)**, glycine **(C)**, histamine **(D)**, and serotonin **(E)** neurotransmitters. *p < 0.05, **p < 0.01; n = 6.

Through an extensive search, we obtained gut microbes associated with neurotransmitter synthesis (Supplementary Table 1). The total abundance of bacteria associated with dopamine, GABA, glycine, histamine, and serotonin (5-HT) synthesis were calculated. We found that microbes synthesize dopamine, GABA and glycine were considerably affected by EEO treatment. EEO significantly increased the abundance of GABA- and glycine-synthesize bacteria in the mouse gut (Figures 8B, C, *P* < 0.05), while it had no significant effect on the abundance of bacteria synthesize histamine and 5-HT (Figures 8D, E, *P* > 0.05). Diazepam treatment significantly increased the abundance of bacteria associated with the synthesis of dopamine, GABA and glycine (Figures 8A–C, *P* < 0.05).

## Discussion

Sleep disorders can seriously affect people quality of life and increase socio-economic burden (Yuen and Pelayo, 2017; Wickwire et al., 2016). In recent years, many studies have explored the effects of natural products on sleep with a potential to replace conventional hypnotic drugs that may have serious side effects. In this study, we explored the sedative-hypnotic effects of EEO treatment in mice. To our knowledge, this is the first report of a depressant effect of EEO on the central nervous system in mice. The open field test was employed to assess whether EEO affected exploratory activity in mice, after the sleep induction test was performed, which was a specific test to assess sedative-hypnotic drugs. In the open field test, it was found that EEO treatment significantly reduced the total distance travelled and decreased the movement speed in mice, and the results of the barbiturate-induced sleep test also found that EEO significantly increased sleep duration. This was consistent with the effects induced by the positive control diazepam treatment group (Figure 1). This suggested that EEO had a significant sedative-hypnotic effect. Consistent with our study, one of the EEO constituents, 1,8-eudesmusin, was reported to reduce locomotion in mice with sedative effect (Santos and Rao, 2000). The main components of EEO were shown in Supplementary Table 2, and one of its main components, 1,8-eudesmusin, may be the main functional substance that exerted sedative-hypnotic effects. In terms of the safety of EEO, a study found that mice did not show significant changes in behavior, vital signs, and blood biochemical indicators when orally administered with 833 mg/kg B.W. of EEO (Kapnisis et al., 2022). According to the World Health Organization, this may indicate that EEO is classified as “unlikely to cause any acute or subacute hazard.” The good sedative-hypnotic effects of EEO make it a safe natural extract that has the potential to be used as an alternative to traditional hypnotic drugs for improving sleep.

Sleep regulation is a highly complex process that is finely regulated by changes in brain neurochemicals, and many neurotransmitters are involved in sleep regulation. Drugs that interfere or modulate these neurochemical systems can lead to changes in arousal and sleep, which in turn modulate sleep disorders. GABA is produced from glutamate by the enzyme glutamic acid decarboxylase. GABA is the main inhibitory neurotransmitter and it is well established that GABA inhibits nerve conduction and prevents neuronal over-excitation which in turn promotes sleep (Yamatsu et al., 2016; Kim et al., 2019). Glutamate is an excitatory neurotransmitter, and glutamate and glutamine can be interconverted, and glutamate can be inactivated by the glutamate-glutamine cycle, terminating the excitatory effect of glutamate (Chen et al., 2022). The results of our study showed that both EEO and diazepam groups significantly increased the levels of glutamine and GABA in the brain of mice compared to the control group (Figure 3). Similar to our study, many natural products such as *Nelumbo nucifera* Seed Extract and Valerian/Hop Mixture can improve sleep and prolong sleep duration in *Drosophila* by modulating GABA signal (Jo et al., 2018; Choi et al., 2017). Our study also found that EEO significantly increased glycine levels in the mouse brain (Figure 3). In the central nervous system, glycine acts as an inhibitory neurotransmitter by affecting glycine receptor GlyRs, and glycine has been reported to increase the duration of non-rapid eye movement sleep and reduce the number and mean duration of dark period awakening (Hondo et al., 2011). An increasing number of studies have found that 5-HT promotes sleep (Zhang et al., 2018), and tryptophan, as the sole substrate for 5-HT synthesis, have also been reported to have a hypnotic effect (Roth et al., 2021). N-acetylserotonin and 5-HIAA, as products of 5-HT acetylation and monoamine oxidation, have also been reported to improve sleep. The results of the study found that EEO could upregulate the levels of tryptophan, N-acetylserotonin and 5-HIAA in brain tissues (Figure 3). The study by Wang et al. (2020) found that Schisandrin B could reduce sleep latency in parachlorophenylalanine-induced insomnia in rats by increasing the levels of 5-HIAA and increasing sleep duration. Nyctinastic herbs decoction can also treat insomnia by increasing 5-HIAA levels (Yang et al., 2021). Supplementation with saffron extract increased N-acetylserotonin and tryptophan synthesis and improved sleep quality in rats (Munoz et al., 2023). Our studies illustrated the ability of EEO to affect the levels of sleep-related neurotransmitters in the brain, which in turn affected the sleep state.

Studies in recent years have found a strong association between gut microbes and sleep, and maintaining the homeostasis of the gut microbes is key to improving sleep (Neroni et al., 2021). Intake of *Lactobacillus* and *Bacillus longum* has been reported to improve sleep quality preventing sleep disorders (Matsuda et al., 2020). A study by Guo et al. (2019) found that Oolong Tea Polyphenols regulate circadian rhythms by enhancing beneficial gut microbes. All these findings suggested that gut microbes influenced sleep quality through GMBA. In our study, we found that EEO and the positive control diazepam significantly altered the gut microbes of mice and increased the α-diversity and β-diversity of the gut microbes (Figure 4). And observed-species and chao1 index showed significantly negative correlation with both total distance and movement speed. Shannon and simpson index showed significantly negative correlation with movement speed and total distance, respectively (Figure 7A). In addition, the gut microbes of mice in the EEO and diazepam groups possessed a higher number of repetitive OTUs and had a more similar flora structure. It is reported that gut microbial diversity is positively correlated with an increase in sleep efficiency and total sleep time, and that gut microbial diversity promotes healthier sleep (Smith et al., 2019). A study by Ogawa et al. (2020) found that treatment with a broad-spectrum antibiotic significantly reduced the duration of non-rapid eye movement sleep in mice while decreasing their gut microbes. These suggested that the diversity of gut microbes affected sleep quality. The diversity of gut microbes interacted with sleep conditions, and poorer sleep states reduced the diversity of gut microbes, leading to dysbiosis. A good gut microbial ecology promoted better sleep. This might be related to the fact that certain beneficial flora promoted sleep, or it might be due to the fact that gut microbes metabolism affected the synthesis of certain neurotransmitters and certain vitamins associated with sleep (El Aidy et al., 2020).

We found that EEO treatment altered the relative abundance of gut microbes, significantly increased the relative abundance of Firmicutes, Proteobacteria, and Actinobacteria at the phylum level and decreased the Bacteroidota/Firmicutes abundance ratio. At the genus level, EEO treatments increased the abundance of *Bacteroides*, *Dubosiella*, *Prevotella_9*, and *Lachnospiraceae_NK4A136_groups* (Figure 5). Lower Bacteroidota/Firmicutes ratio was beneficial for minimising pathogens and for probiotic colonisation (Ge et al., 2023). It has also been previously found that higher Firmicutes abundance is associated with higher sleep quality in the host (Smith et al., 2019). Therefore, we speculated that EEO may affect the abundance of Firmicutes, promoted probiotic colonisation, maintain intestinal homeostasis and exerted a sedative-hypnotic effect. In addition to this, we found that most of the gut microbes altered by EEO were short-chain fatty acid-producing beneficial bacteria. The members of Firmicutes mainly included beneficial bacteria, and Firmicutes were considered to be the main gate of short-chain fatty acid production in the mouse gut (Li W. T. et al., 2023; Wongsurawat et al., 2023). Proteobacteria promoted propionic acid production, and Actinobacteria mainly produced acetic acid and butyric acid (Zhao et al., 2019; Haenen et al., 2013). *Bacteroides* have been shown to be carbohydrate degraders and short chain fatty acid (SCFA) producers (Fan et al., 2023). SCFA produced by gut microbes were thought to be endogenous circadian signal molecules and were essential for gut-brain axis communication (Fredericks et al., 2020). SCFA interacted with receptors on enteroendocrine cells by inducing the secretion of gut hormones, such as GABA and 5-HT, and facilitating indirect signals to the brain via the somatic or vagal pathways (Silva et al., 2020), which can in turn alter the organism’s sleep state. Therefore, EEO may improve sleep by enriching SCFA-producing microbes and thereby increasing the metabolite SCFA.

Compositions of gut microbes were closely related to sleep quality, and we found that sleep duration time showed a significant correlation with the relative abundance of certain gut microbes using the Pearson correlation coefficient test. At the phylum level, Proteobacteria showed a significantly positive correlation with sleep duration (Figure 7B). Similar to our study, Song et al. (2022) found that the shorter the sleep duration caused by caffeine, the lower the relative abundance of Proteobacteria. At the genus level, *Enterococcus*, *Pseudomonas*, *Cupriavidus*, *Prevotella_9*, and *Ralstonia* showed significantly positive correlation with sleep duration (Figure 7C). Insomnia caused a decrease in *Prevotella* abundance in the gut, while the Radix Ginseng and Semen Ziziphi Spinosae treatment improved insomnia and was able to increase *Prevotella* abundance in the gut (Qiao et al., 2022). All of these studies suggested a strong association between sleep and certain microbes. Although there was insufficient evidence to prove a clear causal relationship between sleep problems and microbiological composition, it is undeniable that modulation of the gut microbes can be a key target for the treatment of sleep disorders.

Studies have now demonstrated that the gut microbes can communicate with the brain through functional metabolites, which in turn control circadian rhythms and sleep quality (Ogawa et al., 2020). Differential microbes in the prediction of KEGG function in gut microbes may be related to glutamine and glutamate metabolism (Figure 6D), associated with this, while there are elevated levels of glutamine in the brain. Therefore, we speculated that there may be gut microbes-induced neurotransmitter differences in the brain. In addition, the effect of EEO on the neurotransmitter-producing flora of the gut microbes was explored. Gut microbes were directly involved in the synthesis of a variety of neurotransmitters and can influence the levels of neurotransmitters in the nervous system through metabolic pathways (Zhao et al., 2022). Olson et al. (2018) found that *Parabacteroides* produced large amounts of GABA. *Oscillibacter*, *Dialister*, and *Coprococcus* have been reported to be associated with GABA synthesis (Liu and Huang, 2019). *Bacteroides*, *Prevotella*, *Parabacteriodes* have been reported to be involved in glycine production (Liu and Huang, 2019; Lynch et al., 2017). GABA and glycine, as the main inhibitory neurotransmitters in the nervous system, played an important role in the regulation of the sleep-wake cycle. There was no direct evidence that gut-produced GABA can cross the blood-brain barrier (BBB), but studies have found that physiological and psychological stress can increase BBB permeability and cause GABA inward flow (Constans et al., 2020; Varanoske et al., 2022). In contrast, glycine readily crossed the BBB via glycine transporter proteins and enters the brain to act on NMDA receptors in the supraoptic nucleus, which in turn affected the sleep cycle (Epping et al., 2022). Our finding that EEO significantly increased the abundance of gut microbes associated with GABA and glycine synthesis (Figures 8B, C), and EEO significantly increased GABA and glycine levels in the mouse brain (Figure 3B). The mechanism by which EEO regulates neurotransmitters in the brain is unclear, whether it acts directly on the brain to affect neurotransmitter synthesis or regulates neurotransmitter levels via gut microbes will require further studies to elucidate the exact mechanism. However, it is well known that gut microbes produce hormones and neurotransmitters that are transmitted via the afferents of the vagus nervous system, which activate or inhibit the corresponding neurons, thereby promoting sleep (Lyte, 2014; Dinan et al., 2015).

## Conclusion

EEO supplementation reduced activity and improved sleep quantity in mice, consistent with the effects of diazepam. Through brain neurotransmitter-targeting histological assays, EEO was shown to increase brain levels of sleep-promoting neurotransmitters such as glutamine, GABA, glycine, tryptophan, N-acetylserotonin and 5-HIAA, and improve sleep in mice. EEO increased the abundance of intestinal flora, short-chain fatty acid-producing flora, and promoted probiotic colonisation, as well as the abundance of functional flora synthesize GABA and glycine neurotransmitters. EEO also increased the abundance of short-chain fatty acid-producing flora and the abundance of functional flora synthesizing GABA and glycine neurotransmitters. EEO may exert sedative-hypnotic effects by improving the gut microbiota and thereby altering brain neurotransmitters. Our findings suggested that gut microbes might serve as a target of action for EEO in the treatment of sleep deprivation, EEO as a natural product alternative for the treatment of insomnia and sleep disorders, and the potential for the development of new drugs for the treatment of sleep disorders.

## Data Availability

The original contributions presented in the study are included in the article/Supplementary Material, further inquiries can be directed to the corresponding author.
