# Supplementary material for: Eucalyptus essential oil exerted a sedative-hypnotic effect by influencing brain neurotransmitters and gut microbes via the gut microbiota-brain axis
Source: Front Pharmacol. 2024 Sep 25;15:1464654. doi: 10.3389/fphar.2024.1464654 (PMC11461282; doi:10.3389/fphar.2024.1464654)
Supplement: Supplementary file 1 [file Table1.pdf]

Table S1. The gut microbes in genus levels related to neurotransmitter synthesis.

Neurotransmitter	Neurotransmitter Producing bacteria	DOI
Serotonin	*Bacteroides*<br>*Escherichia*<br>*Hafnia*<br>*Lactobacillus*<br>*Coprococcus*	https://doi.org/10.1007/978-3-030-32962-4_22<br>https://doi.org/10.1016/j.brainres.2018.03.015
Dopamine	*Bacillus*<br>*Clostridium*<br>*Yersinia*<br>*Salmonella*<br>*Morganella*	https://doi.org/10.1007/978-3-030-32962-4_22<br>https://doi.org/10.1016/j.brainres.2018.03.015
GABA	*Oscillibacter*<br>*Dialister*<br>*Coprococcus*<br>*Parabacteroides*	https://doi.org/10.1007/978-3-030-32962-4_22<br>https://doi.org/10.1016/j.brainres.2018.03.015
Glycine	*Bacteroides*<br>*Prevotella*<br>*Parabacteriodes*	https://doi.org/10.1007/978-3-030-32962-4_22<br>https://doi.org/10.1016/j.brainres.2018.03.015
Histamine	*Bacteroides*<br>*Lactobacillus*	https://doi.org/10.1007/978-3-030-32962-4_22<br>https://doi.org/10.1016/j.brainres.2018.03.015

Table S2. Main components and content of EEO

Compounds	Class I	CAS	Relative Content %
8-Eucalyptin	Terpenoids	470-82-6	7.422
1,4-Eucalyptin	Terpenoids	470-67-7	7.422
(S)-2,5-Dimethyl-3-vinylhex-4-en-2-ol	Alcohol	35671-15-9	6.357
Benzyl Alcohol	Alcohol	100-51-6	6.247
(E)-3-Octen-2-one	Ketone	18402-82-9	5.158
Pantolactone	Ester	599-04-2	4.308
Cyclohexanol, 3,5-dimethyl	Alcohol	5441-52-1	4.308
β-Phellandrene	Terpenoids	555-10-2	3.937
(Z)-1,3,6-Octatriene, 3,7-dimethyl	Terpenoids	3338-55-4	3.935
β-Ocimene	Terpenoids	13877-91-3	3.935
2-Butenoic acid, butyl ester	Ester	7299-91-4	3.163
3-Cyclohexen-1-one,3,5,5-trimethyl	Ketone	471-01-2	3.121
2-Azabicyclo[3.2.1]octan-3-one	Ketone	26396-67-8	3.116
2-Furancarboxylic acid, ethyl ester	Heterocyclic compound	614-99-3	1.889
Limonene	Terpenoids	138-86-3	1.887
Cyclohexene, 1-methyl-4-(S)-1-methylethenyl	Terpenoids	5989-54-8	1.886
1,5-Cyclooctadiene, 3,4-dimethyl	Hydrocarbons	21284-05-9	1.881
o-Cymene	Terpenoids	527-84-4	1.862

Benzene, 1-methyl-3-(1-methylethyl)	Terpenoids	535-77-3	1.862
3-Octen-2-one	Ketone	1669-44-9	1.725
1,3-Hexadiene, 3-ethyl-2-methyl	Hydrocarbons	61142-36-7	1.679
Acetic acid, cyclohexyl ester	Ester	622-45-7	1.677
Thiazole, 5-ethenyl-4-methyl	Heterocyclic compound	1759-28-0	1.123
